# The emergence of idiosyncratic patterns in the frequency-following response during the first year of life

**DOI:** 10.1121/10.0010493

**Published:** 2022-05-10

**Authors:** Fernando Llanos, T. Christina Zhao, Patricia K. Kuhl, Bharath Chandrasekaran

**Affiliations:** 1 Department of Linguistics, University of Texas at Austin, Austin, Texas 78712, USA; 2 Department of Speech and Hearing Sciences, University of Washington, Seattle, Washington 98195, USA; 3 Institute for Learning and Brain Sciences, University of Washington, Seattle, Washington 98195, USA; 4 Department of Communication Sciences and Disorders, University of Pittsburgh, Pittsburgh, Pennsylvania 15260, USA fllanos@utexas.edu, zhaotc@uw.edu, pkkuhl@uw.edu, b.chandra@pitt.edu

## Abstract

The frequency-following response (FFR) is a scalp-recorded signal that reflects phase-locked activity from neurons across the auditory system. In addition to capturing information about sounds, the FFR conveys biometric information, reflecting individual differences in auditory processing. To investigate the development of FFR biometric patterns, we trained a pattern recognition model to recognize infants (*N* = 16) from FFRs collected at 7 and 11 months. Model recognition scores were used to index the robustness of FFR biometric patterns at each time. Results showed better recognition scores at 11 months, demonstrating the emergence of robust FFR idiosyncratic patterns during this first year of life.

## 1. Introduction

**Fig. 1.**
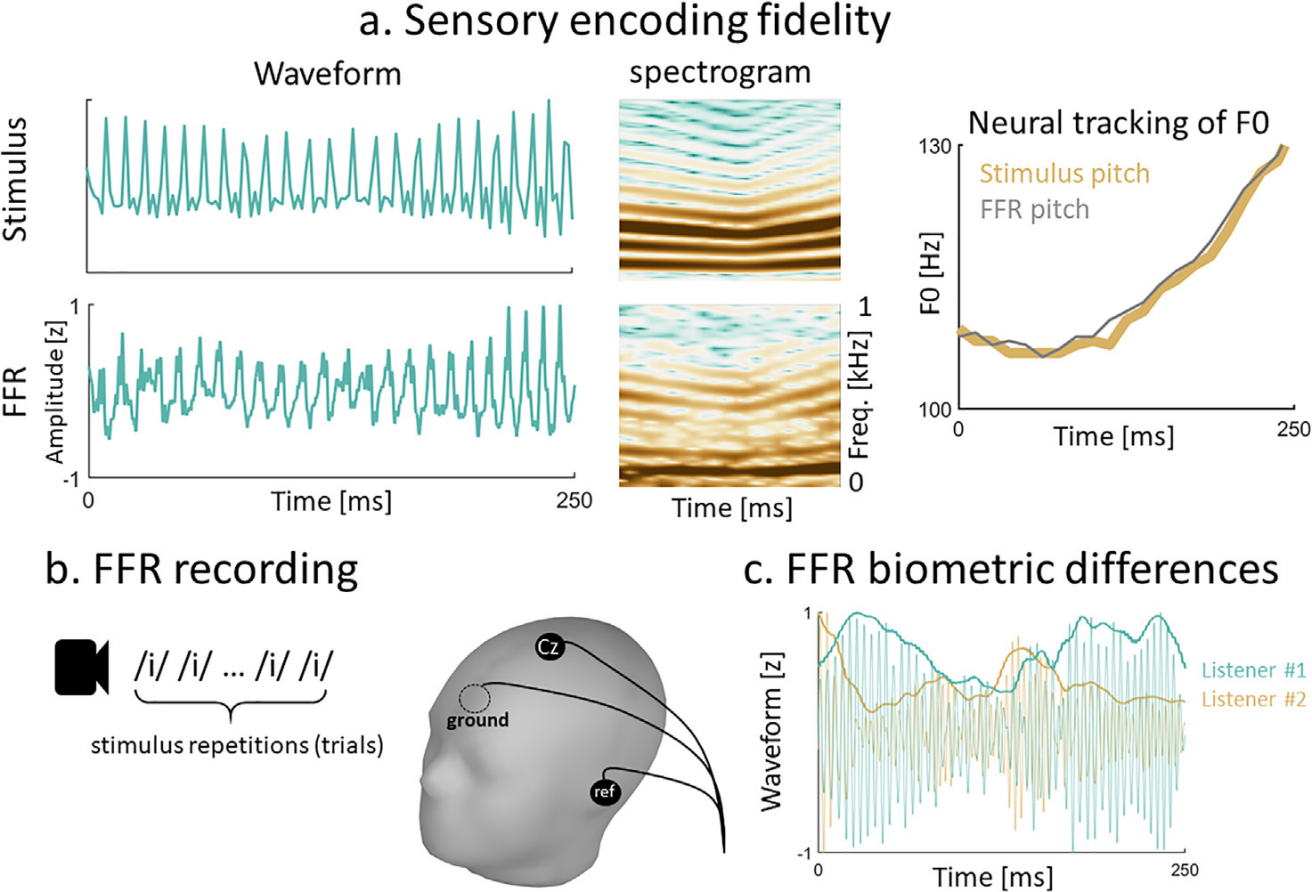
The FFR. (a) Similarity between the waveforms, spectrograms, and F0 contours of the evoking stimulus used in the present study and its FFR. (b) The FFR is usually collected with three scalp-electrodes while participants are listening to thousands of stimulus repetitions. (c) The FFR contains spectro-temporal patterns that vary across listeners. This property is illustrated with the FFRs of two listeners. FFRs were bandpass filtered between 180 and 280 Hz, right above the F0 range of the evoking stimulus. Waveform amplitudes were normalized for visualization purposes.

During the first year of life, infants undergo a series of neurodevelopmental changes in auditory sensory processing that result from auditory experience and the maturation of the auditory system (Eggermont and Moore, 2012; Werner and Marean, 2019). Because infants lack the cognitive maturity that is required to perform in most behavioral tasks, recent electroencephalography (EEG) work has leveraged the frequency-following response (FFR) to investigate these neurodevelopmental changes (Anderson *et al.*, 2015; Jeng *et al.*, 2010; Lemos *et al.*, 2021; Skoe *et al.*, 2015). The FFR is a far-field auditory-evoked potential that does not require attention to be observed (Coffey *et al.*, 2019; Krizman and Kraus, 2019; Skoe and Kraus, 2010). The FFR reflects sustained phase-locked activity from multiple nuclei in the auditory pathway and primary auditory cortex. When the central auditory system is stimulated with a periodic waveform, these nuclei synchronize their oscillatory activity by firing in phase at each cycle of the waveform. This synchronized activity, which is aggregated by the FFR, provides a sensory representation of the temporal structure of the stimulus signal with a high degree of fidelity [Fig. 1(a)]. Using the FFR, prior infant work has documented the early maturation of neural mechanisms that are critical for the neural representation of sound, such as neural phase-locking (Van Dyke *et al.*, 2017).

The representation of sound features like the fundamental frequency (F0) in the FFR is influenced by experience (Krishnan *et al.*, 2010; Reetzke *et al.*, 2018; Wong *et al.*, 2007). For example, the FFRs of native speakers of languages that are tonal (e.g., Mandarin Chinese) and musicians exhibit more robust tracking of stimulus F0 than the FFRs of native speakers of languages that are not tonal and non-musicians (Bidelman *et al.*, 2011). These findings indicate that the FFR captures plasticity driven by long-term auditory experiences, including bilingualism (Bsharat-Maalouf and Karawani, 2022). In this body of literature, the neural coding of F0 is evaluated by comparing the F0 contours of the FFR and evoking stimulus in the temporal or spectral domain [e.g., Fig. 1(a)].

The FFR also conveys biometric patterns [Fig. 1(c)] that account for individual differences in neural processing (Coffey *et al.*, 2016; Kraus *et al.*, 2016; Reis *et al.*, 2021) and can be used to identify and discriminate between listeners (Llanos *et al.*, 2019; Xie *et al.*, 2017). In a previous study (Llanos *et al.*, 2019), we used a pattern recognition model to recognize adult listeners by their FFRs. Crucially, the performance of the model remained robust even when the FFR was averaged across a small number of trials or stimulus repetitions. The small averaging size required to recognize listeners was a major finding because the number of trials that is required to evaluate F0 encoding in most FFR studies exceeds 1000. Thus, our approach provided a potential method to investigate the development of biometric patterns in the FFRs of infants, who usually struggle to engage in long experimental sessions. In the present study, we extended the methodologies developed in Llanos *et al.* (2019) to investigate the development of FFR idiosyncratic patterns (spectro-temporal FFR patterns that can be used to identify and discriminate between listeners).

We tested the hypothesis that the early maturation of the auditory system combined with enriched auditory experience would result in more robust biometric profiles, operationally defined as individual FFR morphologies that are easier to recognize. To evaluate this hypothesis, we trained a hidden Markov model (HMM; Llanos *et al.*, 2017, 2019) to recognize infants by their FFRs and used the recognition scores (the log-probability of being recognized by the model) to index the amount of biometric information encoded in the FFR of each infant. FFRs were longitudinally collected at 7 and 11 months of age because during this period infants' auditory perception is strongly influenced by auditory experience (Bosseler *et al.*, 2013; Maurer and Werker, 2014; Werker and Tees, 1984). To evaluate the effects of this period on FFR biometrics, we compared infant recognition scores between sessions.

As we note above, one experimental limitation of the FFR is the large number of trials that is usually required for averaging purposes (Skoe and Kraus, 2010). Because infants cannot always participate in long EEG sessions and their EEGs are often contaminated with movement artifacts that are hard to suppress, this experimental limitation has contributed to the lack of infant data in this area. To that end, we investigated the effects of averaging size on the performance of the model. We also manipulated the range of FFR frequencies used to recognize infants to assess the brain oscillation bands that encoded more robust biometric patterns. To optimize the performance of the model, individual bands were averaged across the number of trials providing higher recognition scores.

Finally, we leveraged two well-established metrics of neural F0 encoding to assess the sensory representation of stimulus pitch at each session: neural F0-strength and stimulus-response correlation (Krishnan *et al.*, 2010; Reetzke *et al.*, 2018). Neural F0-strength measures how much stimulus periodicity is preserved in the FFR. Stimulus-response correlation evaluates the representation of F0 kinematics in the FFR. We focused on these metrics because they have been shown to capture plasticity driven by auditory experience (Krishnan *et al.*, 2010; Reetzke *et al.*, 2018). We compared the results of these metrics, which require larger FFR averaging sizes (>1000 trials) than the HMM (Llanos *et al.*, 2017, 2019), to determine which of these approaches was able to capture more robust developmental changes in the FFR.

## 2. Methods

### 2.1 Participants and EEG acquisition

All experimental procedures were approved by the University of Washington Institutional Review Board. Infants were recruited from monolingual English households [family standard effect size (SES): mean (M) = 53.64, standard deviation (SD) = 8.90 Hollingshead scale]. The inclusion criteria included the following: (a) born within 14 days of the due date; (b) no more than three ear infections and no known health problems; (c) birth weight ranging from 6 to 10 lb. Ten infants reported to have some level (M = 5.80 h/month, SD = 7.07) of foreign language exposure (music and library story time). Average music exposure (e.g., parents singing and radio) was 20.57 h/week (SD = 13.44) at 7 months and 26.35 h/week (SD = 24.51) at 11 months. Two of the 26 infants who were tested at 7 months (age: M = 27.27 weeks, SD = 0.94; 14 males) became too fussy before data recording. Twenty infants returned for testing at 11 months (age: M = 48.56 weeks, SD = 1.22; 10 male). Of these, two become very fussy before the EEG session. Only infants with more than 1500 trials accepted at both sessions were included in the final analyses (*N* = 16).

During each session (7 vs 11 months), infants were exposed to 3000 repetitions of one synthesized high-front vowel bearing a low-dipping F0 contour like the one shown in Fig. 1(a) (105 Hz–89 Hz–110 Hz), which has been demonstrated to elicit better signal quality than flat or falling F0 (first formant = 330 Hz; second formant = 2500 Hz; F0 dipping from 101 to 87 and 107 Hz) (Llanos *et al.*, 2017; Reetzke *et al.*, 2018). EEGs were collected with three electrodes placed on the vertex (Cz, active), right earlobe (reference), and forehead (ground). EEG signals were digitized and amplified in a NeuroScan system at 20 kHz. The audio was presented monaurally via one insert earphone to the right ear at 75 dB sound pressure level (SPL). The inter-stimulus interval was jittered around 300 ± 20 ms, and the polarity of the stimulus waveform was alternated across trials to attenuate the cochlear microphonics effect. The whole experimental session lasted approximately 45 min, with approximately 15 min of recording. Breaks were taken when infants became fussy or when electrodes needed to be adjusted. For every infant, we were able to collect a minimum of 1500 artifact-free trials with an amplitude smaller than ±40 μV relative to the mean amplitude of the 50 ms portion of the EEG preceding the onset of the auditory stimulus.

### 2.2 FFR biometrics

#### 2.2.1 HMM procedures

The HMM was defined as a chain of three hidden states feedforward connected in steps of one and two (Llanos *et al.*, 2017, 2019; Reetzke *et al.*, 2018). We trained a total of 128 HMMs (16 infants × 2 sessions × 4 averaging sizes). FFR averaging size ranged from the optimal value reported in Llanos *et al.* (2019) (*N* = 200 trials) to three small values (*N* = 1, 5, and 50 trials) selected from Llanos *et al.* (2019) to explore the performance of the HMM at low signal-to-noise ratio (SNR) levels. Each HMM was trained to recognize the FFRs of one infant in one session. Training and testing parameters were adopted from Llanos *et al.* (2019) (codebook size = 150 Voronoi cells, training size = 750 trials, cross-validation method = k-fold). To manipulate the SNR of FFR, training and testing sets were sub-averaged with a moving average window that combined trials evoked with different stimulus polarities. Each sub-averaged FFR was divided into a sequence of fast Fourier transform (FFT) spectrum slices (slice length = 20 ms, overlap = 15 ms). Each FFT sequence was converted into a sequence of discrete symbols, or emissions, using the vector quantization procedures described in Llanos *et al.* (2019). During the training phase, the HMM was trained to generalize a stochastic representation of the emission sequences included in the training set. During the testing phase, we used the log-probability metric (Llanos *et al.*, 2019) to quantify the degree of similarity between each emission sequence in the testing set and the stochastic representation generalized by the model during the training phase. Log-probabilities were averaged into one single recognition score per model to reduce error type II in further statistical analysis.

#### 2.2.2 model reliability

To assess the reliability of the model, we examined the extent to which infant recognition scores were above the empirical level of chance. The empirical level of chance for each HMM was determined via permutation test (Anderson and Braak, 2003; Xie *et al.*, 2019). We shuffled FFR trials across infants and followed the training and testing procedures introduced in Sec. 2.2.1 to derive one *chance-level* recognition score for each HMM. We repeated these steps multiple times to create a distribution of 100 chance-level recognition scores for each HMM. Next, we averaged the chance-level recognition scores by session and averaging size and calculated the proportion of them that were equal to or higher than the average of the corresponding *ground truth* scores obtained as in Sec. 2.2.1. We used this proportion as a *p*-value to reject the null hypothesis that the HMM was performing at chance in the corresponding session and averaging size.

#### 2.2.3 Effects of session on infant recognition scores

**Fig. 2.**
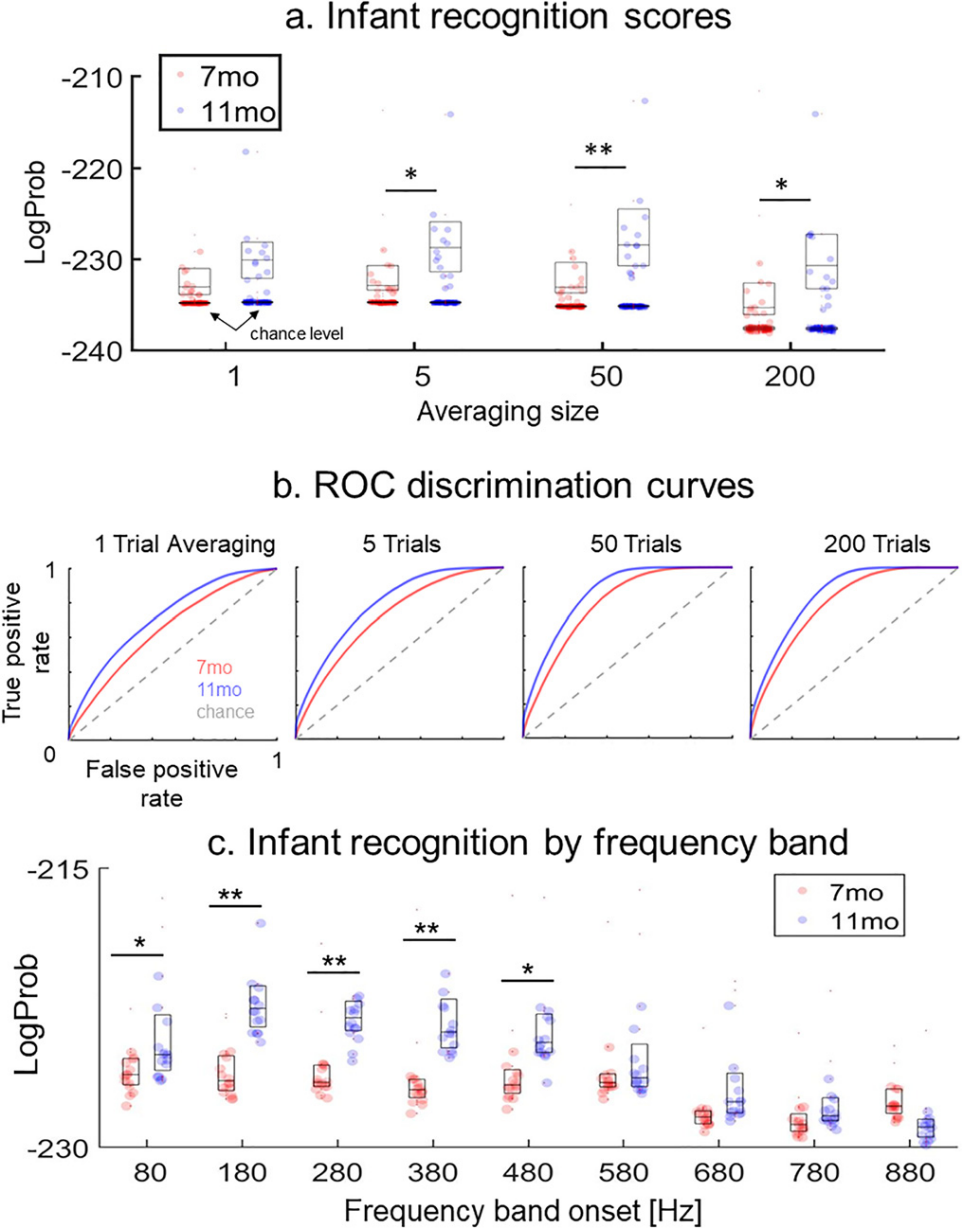
FFR biometric results. (a) Individual infant recognition scores organized by averaging size and session. Boxplot horizontal lines represent median and percentiles. The darker dots below the boxplots represent the chance-level recognition scores derived from the permutation analysis. Asterisks denote relative differences in statistical significance. (b) ROC curves organized by session and averaging size. (c) Recognition scores organized by session and frequency band (averaging size = 50 trials). In the *x* axes, the frequency band is denoted by the onset frequency of the band (e.g., 80 means 80–180 Hz). Asterisks denote relative differences in statistical significance. The *p*-values are provided in the text. Outlier dots (>90%) are not shown in the boxplot, but they were included in the statistical analyses.

Infant recognition scores were modeled with the following linear mixed-effects equation in R (*lme4* package): *score ∼ session + averaging_size + session:averaging_size + (1|infant)*. The equation incorporated fixed effects and interactions by session (reference level = 7 months) and averaging size and random intercepts by infant. The reference level for averaging size was set to the size providing higher recognition scores [*N* = 50 trials; see Fig. 2(a)]. To assess the effects of session by averaging size on non-reference levels, we conducted *post hoc* Tukey-adjusted pairwise comparisons in R (*emmeans* package).

#### 2.2.4 Discrimination performance

To diagnose the discrimination performance of the HMM, we examined the receiver operating characteristic (ROC) curve of the model for each averaging size in each session. The ROC curve is a standard metric of classification performance in machine learning and data mining work, including machine learning studies with a focus on clinical diagnosis (Linden, 2006). The ROC curve represents the trade-off between the true and false positive rate of a classifier across multiple discrimination thresholds and thus provides a big picture of its discrimination performance. The discrimination performance of a recognition model can be ranked into one of five categories as a function of the area under the ROC curve: excellent (area > 0.9), good (>0.8), fair (>0.7), poor (>0.6), or bad (>0.5) (Carter *et al.*, 2016). To calculate the ROC of the model for each averaging size in each session, we used the HMMs trained in Sec. 2.2.1 to discriminate between emission sequences from different infants. Following this approach, each emission sequence in each testing set was classified into the infant category of the HMM providing the largest log-probability (i.e., greatest recognition score). ROC curves were averaged by session and averaging size and ranked into one of the five categories introduced above as a function of their area. We also calculated the percentage of emission sequences that were classified into the right infant category (i.e., discrimination accuracy) for each averaging size in each session.

#### 2.2.5 Optimal recognition by frequency band

We examined the frequency bands of the FFR that provided better recognition scores. To optimize the performance of the model, FFR frequency bands were averaged across the number of trials providing the highest infant recognition scores (*N* = 50 trials). We applied the training and testing procedures introduced in Sec. 2.2.1 to different frequency channels extracted from the FFT. For each frequency channel, we selected the FFT values (converted to dB) between the upper and lower frequency bounds of the channel. The frequency bands of the channels ranged from 80 to 980 Hz in steps of 100 Hz (Llanos *et al.*, 2019). Infant recognition scores across frequency bands were modeled with the following linear mixed-effects equation in R: *score ∼ session + band + session:band + (1|infant)*. The equation incorporated fixed effects by session (reference level = 7 months) and frequency band, their interaction, and random intercepts by infant. The reference for frequency band was set to a level providing better recognition scores [180–280 Hz; see Fig. 2(c)]. To assess the effects of session for each band, we conducted *post hoc* Tukey-adjusted pairwise comparisons in R (*emmeans* package).

### 2.3 Neural F0 encoding metrics

Responses were averaged across the 1500 artifact-free trials for each infant in each session. Neural F0 tracking was computed as the Pearson's *r* correlation coefficient between the F0 contours of the averaged response and evoking stimulus (Reetzke *et al.*, 2018). Neural and stimulus F0 contours were extracted with the autocorrelation method using a sliding window of 40 ms duration and 30 ms overlap. Neural F0-strength was determined as the mean autocorrelation peak across sliding windows in the FFR (Reetzke *et al.*, 2018). To assess the SNR of the FFR for each infant in each session, we computed the root mean square (rms) intensity of the averaged response (0–250 ms) over the rms of the pre-stimulus portion of the EEG (−50 to 0 ms).

## 3 Results

### 3.1 FFR biometrics

First, we evaluated the reliability of the model across sessions and averaging sizes. The permutation tests showed that the distributions of recognition scores in each session and averaging size were above the level of chance (*p*-values < 0.001). This indicates that the performance of the HMM was robust across averaging sizes and sessions. Chance-level recognition scores are depicted in Fig. 2(a) (darker dots below each boxplot).

Next, we examined the effects of session and averaging size on infant recognition scores [boxplots in Fig. 2(a)]. The results of the linear mixed-effects model conveyed a significant effect by session (β = 8.23, *z* = 2.84, *p* = 0.005). This indicates that, when the averaging size was fixed to the reference level (50 trials), infant recognition scores at 11 months were better than those at 7 months. The results of the *post hoc* analysis for non-reference levels yielded a significant effect of session (11 > 7 months) for averaging sizes equal to or larger than five trials: one trial: *t*(119) = −1.53, *p* = 0.12; five trials: *t*(119) = −2.22, *p* = 0.02; 50 trials: *t*(119) = −2.75, *p* = 0.006; 200 trials: *t*(119)= −2.72, *p* = 0.007. On the other hand, the fixed effects of averaging size were not significant (*p*-values > 0.36). This indicates that infant recognition scores at 7 months (reference session) did not significantly change from the reference size (50 trials). Further *post hoc* analysis via Tukey-adjusted pairwise comparisons extended this null result to the 11 month session (*p*-values > 0.43). Finally, the interactions between fixed effects in the mixed-effects model were negative but not significant (11 month × 1 trial: *β* = −3.65, *z* = −0.89, *p* = 0.3; 11 month × 5 trials: β = −1.57, *z* = −0.38, *p* = 0.7; 11 month × 200 trials: *β* = −0.07, *z* = −0.01, *p* = 0.9). This indicates that while the effect of session on each averaging size was smaller than on the reference size, these differences were not significant.

Next, we examined the discrimination performance of the model. We found ROC areas in the poor range (one trial at 7 and 11 months and five trials at 7 months), good range (50 and 200 trials at 11 months), and fair range (the other ones). We did not find any ROC area in the excellent (ROC > 0.9) or bad (ROC ≤ 0.6) range. The percentages of discrimination accuracy (Table 1) were consistent with the results of the ROC analysis. All of them were way above the level of chance (= 6.25%). Combined, these results show that the recognition scores conveyed by the model were associated with a good discrimination performance for averaging sizes larger than or equal to 50 trials in the second session (11 months).

**Table 1.** ROC area and discrimination accuracy by session and averaging size.

Averaging size	1 trial	5 trials	50 trials	200 trials
7 months				
ROC area	0.64 (poor)	0.70 (poor)	0.76 (fair)	0.76 (fair)
Discrimination accuracy (% correct)	14.25	19.30	25.51	27.07
11 months				
ROC area	0.70 (poor)	0.75 (fair)	0.82 (good)	0.81 (good)
Discrimination accuracy (% correct)	22.35	31.90	42.57	36.97

Next, we identified the frequency bands that conveyed more robust biometric patterns. The results of the linear mixed-effects modeling conveyed several significant fixed effects and interactions [Table 2 and Fig. 2(c)]. The fixed effects indicated that most bands higher than 580 Hz provided less robust biometric information than the optimal band (180–280 Hz) at 7 months. The interactions showed that the effects of session on the stimulus F0 band (80–180 Hz) and bands above 480 Hz were smaller than in the optimal band. Consistent with this finding, the results of the *post hoc* analysis of band-by-session showed that at 11 months, the optimal band conveyed more biometric information than these other bands: band onset = 80 Hz: *t*(290) = 3.24, *p* = 0.035; 580 Hz: *t*(290) = 4.24, *p* = 0.001; 680 Hz: *t*(290) = 5.95, *p* < 0.0001; 780 Hz: *t*(290) = 7.06, *p* < 0.0001; 880 Hz: *t*(290) = 8.18, *p* < 0.0001. Combined, these results show that the bands providing more robust biometric information were between 180 and 480 Hz. The results of the *post hoc* analysis of session-by-band revealed significant differences between sessions for all bands below 580 Hz (band onset = 80 Hz: *t*(290) = −2.06, *p* = 0.039; 180 Hz: *t*(290) = −5.36, *p* < 0.0001; 280 Hz: *t*(290) = −4.77, *p* < 0.0001; 380 Hz: *t*(290) = −4.85, *p* < 0.0001; 480 Hz: *t*(290) = −3.07, *p* < 0.0001) (bands > 480 Hz: *p*-values > 0.1). These results show that all bands below 580 Hz were able to capture developmental changes.

**Table 2.** Significant effects in the linear mixed-effects modeling of infant recognition scores by band and session. ^*^, *p* ≤ 0.05; ^**^, *p* ≤ 0.01; ^***^, *p* ≤ 0.001. df, degrees of freedom.

	β-coefficient	df	*z*-score	pval
(Intercept)	−225.93	116.10	−287.76	<2e−16^***^
11 months	5.16	272	5.53	7.19e−08^***^
680–780 Hz	−2.15	272	−2.31	0.021^*^
780–880 Hz	−2.46	272	−2.64	0.008^**^
11 months: 80–180 Hz	−3.17	272	−2.40	0.01^*^
11 months: 580–680 Hz	−4.18	272	−3.17	0.001^**^
11 months: 680–780 Hz	−3.57	272	−2.71	0.007^**^
11 months: 780–880 Hz	−4.33	272	−3.28	0.001^**^
11 months: 880–980 Hz	−6.37	272	−4.83	2.25e−06^***^

### 3.2 Neural F0 encoding

**Fig. 3.**
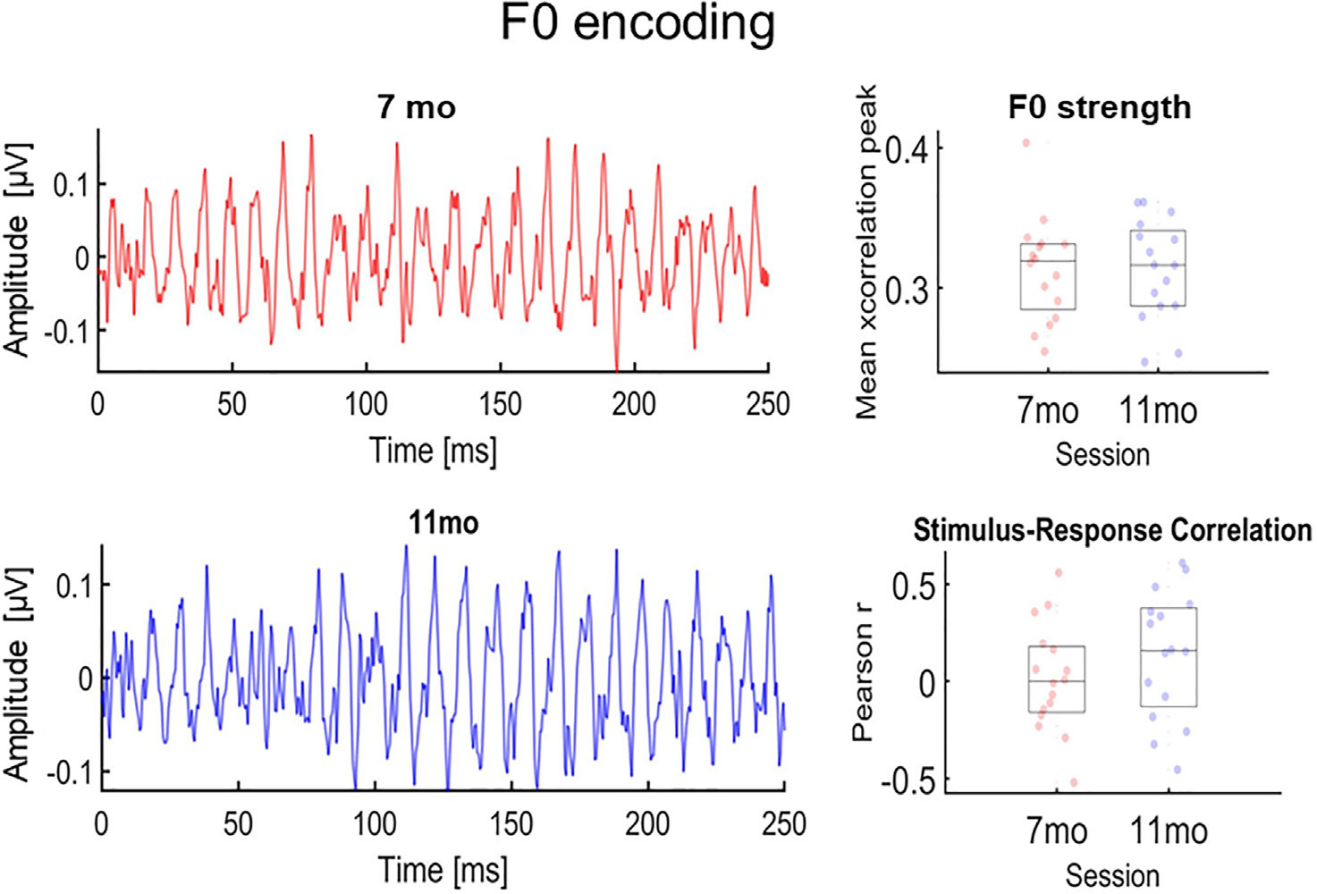
Neural F0 encoding results. FFR waveforms grand-averaged across infants (*N* = 16) by session (left panels). Individual neural F0-strength values (top right panel) and stimulus-response correlation values (bottom right panel) are organized by session. Boxplot horizontal lines represent median and percentiles.

We conducted two two-sample *t*-tests to assess the effects of session (independent variable) on neural F0 tracking and neural F0-strength (dependent variables), and none of the tests provided significant differences between sessions [neural F0 tracking: *t*(15) = 1.03, *p* = 0.31; F0-strength: *t*(15) = −0.03, *p* = 0.97] (Fig. 3). The lack of differences between sessions could be due to the low SNR levels of individual FFRs averaged across 1500 trials (7 month session: M = 1.18, SD = 0.19; 11 month session: M = 1.14; SD = 0.2). The SNR levels observed in the present study were much smaller than the SNR levels that are typically documented for adults in FFR research using even smaller numbers of trials. In this body of literature, SNR levels are usually equal to or higher than 2 for averaging sizes as small as 1000 trials (Llanos *et al.*, 2017; Xie *et al.*, 2017; Reetzke *et al.*, 2018).

## 4. Discussion

We aimed to investigate the development of idiosyncratic FFR patterns during the first year of life. To this end, we trained a pattern recognition model to recognize infants by their FFRs. FFRs were longitudinally collected at 7 and 11 months of age, a period of time during which infants undergo rapid development in many domains that are also interacting with experience (Bosseler *et al.*, 2013; Maurer and Werker, 2014; Diamond, 2006; Werker and Tees, 1984). We used the infant recognition scores provided by the model to index the amount of biometric information encoded in the FFR of each infant in each session.

First, we assessed the performance of the model across FFR averaging sizes and sessions. The model recognized infants by their FFRs with scores way above the level of chance for averaging sizes as small as one single FFR trial. As in prior adult work (Llanos *et al.*, 2017), we did not find significant differences in recognition scores between large averaging sizes (e.g., 50 vs 200 trials). However, our results also did not convey differences between small and large averaging sizes (e.g., 1 vs 50 trials). This unexpected finding suggests that the effects of averaging size on infant recognition scores may emerge later in time.

Discrimination performance was associated with fair (ROC > 0.7) and good (ROC > 0.8) discrimination curves for averaging sizes equal to or above 50 trials. This finding is also consistent with prior adult work (Llanos *et al.*, 2019). However, the discrimination curves reported in this prior work (ROC > 0.9) were better than in the present study. This discrepancy could be due to differences in neural gain between infants and adults. As we noted in Sec. 3.2, the SNR of the FFR for infants was nearly half of the SNR documented for adults in prior FFR work (Llanos *et al.*, 2017). The fact that we were able to recognize infants despite the low SNR indicates that infant biometric patterns were robustly encoded in the neural response.

To assess developmental changes in the amount of biometric information conveyed in the FFR, we examined the effects of session (7 vs 11 months) on infant recognition scores. We hypothesized that the rapid maturation of the auditory system combined with longer periods of meaningful auditory experience would lead to easier-to-recognize individual profiles. This hypothesis was supported by the results. Specifically, we found a robust developmental effect for averaging sizes of five trials and above. This finding demonstrates that pattern recognition approaches are suitable to investigate the emergence and maturation of FFR features across the lifespan. Notably, the oscillation bands in the FFR conveying more robust biometric information were outside the F0 range of the stimulus signal. This finding provides a potential dissociation between the neural encoding of biometric and F0 features in the FFR. This finding is also consistent with prior work (Easwar *et al.*, 2021) showing that infant-adult differences in the FFR are also modulated by frequency band.

Prior FFR work with children has shown that, during the first 10 years of life, the neural encoding of F0 becomes gradually more consistent across trials within the same session (Skoe *et al.*, 2015). While our model was trained to recognize biometric and not F0 features, the higher recognition scores conveyed by the model in the second session indicate that FFR biometric features were more consistently represented across trials at 11 months. Thus, the recognition performance of our model at the older age could have been facilitated by a more consistent encoding of FFR features over time.

In sum, the results of the present study advance our current scientific understanding of the development of individual neural differences during the second half of the first year of life. Critically, our method provides a new angle to investigate these differences in early development when it is difficult to record thousands of trials with high SNR from infants. From a methodological perspective, our method was able to capture robust developmental effects that were not captured by our traditional metrics of neural F0 encoding (F0-strength, stimulus-response correlation), which were less robust against low SNR values. Because the HMM can learn a wide variety of spectro-temporal patterns in an unsupervised manner, future research could leverage this approach to identify FFR-based markers of neurodevelopmental disorders or individual differences in auditory processing across the lifespan.
